# Application of the Harms Technique to Treat Undiagnosed Intractable C1-C2 Unilateral Neck Pain: A Case Report

**DOI:** 10.7759/cureus.793

**Published:** 2016-09-21

**Authors:** Adnan Bashir Bhatti, Sunny Kim

**Affiliations:** 1 Medical Director of Clinical Research, Spine Surgery, Tristate Brain and Spine Institute; 2 Spine Surgery, Tristate Brain and Spine Institute

**Keywords:** harms technique, neck pain, cervical spine, arthrodesis, atlantoaxial, c1-c2

## Abstract

A 79-year-old female presented with incapacitating chronic neck pain. The patient's pain which was greatest on the left side persisted for 18 months and was described as stabbing in nature (10/10 intensity). In addition to her neck pain, the patient described having frequent headaches. After six weeks of physical therapy and undergoing a rhizotomy procedure, she showed no prolonged improvement. An epidural steroid injection provided only temporary pain relief and was followed by a successful posterior fusion using the Harms technique with iliac crest autogenous bone grafting and placement of polyaxial screws in the C1 lateral masses and C2 pedicles. At the one-year follow-up the patient reported no pain or complaints. In general, C1-C2 arthrodesis is a surgical challenge due to the proximity of neurovascular structures (vertebral arteries and spinal cord) and wide range of the joint motion. The Harms technique is one of many techniques developed to reduce anatomical risk and improve results related to biomechanical stability and fusion rates.

## Introduction

Neck pain caused by damaged cervical facet joints is a common cause of disability [1]. Damage to C1-C2 can elicit symptoms that include nerve irritation, vertebrobasilar insufficiency with associated vertigo, tinnitus, dizziness, facial pain, arm pain, and migraine headaches. This diverse range of symptoms can make chronic neck pain a challenge to diagnose and treat [2].

Current diagnostic tools available for neck pain are inconsistent and lack specificity. According to Manchikanti, et al., imaging studies such as magnetic resonance imaging (MRI), computed tomography (CT), radiography, and single-photon emission computed tomography (SPECT) are non-diagnostic for the cervical spine. With the exception of disk herniation and spondylosis, degenerative changes to the cervical spine shown by MRI do not correlate with neck symptoms. However, there is strong evidence to support the diagnostic accuracy of cervical facet joint nerve block when used in conjunction with history findings, physical examinations, and appropriate radiologic workup [3].

In general, initial management of neck pain is conservative (e.g. non-invasive interventions) and can include physical therapy, exercise programs, and drug therapy. However, unlike invasive interventions the application of non-invasive interventions can make it difficult to diagnose neck pain in some patients [3].

An invasive intervention for neck pain management is an atlantoaxial arthrodesis. Atlantoaxial arthrodesis is a technically demanding surgical procedure because of C1-C2 anatomy and the large range of C1-C2 joint motion that can hinder bone fusion [4]. In 1939, Dr. Gallie first described posterior surgical wiring and fusion of the C1-C2, and it was later improved in the 1970s by Dr. Brooks. Nonetheless, a drawback of this technique is limited biomechanical efficacy and real three-dimensional stability [5].

In 1986, the transarticular screw technique, also known as the Magerl technique, which provides a fusion rate of almost 100% was introduced. However, the Magerl technique still requires preliminary reduction of C1-C2 before screw placement. Additionally, the Magerl technique requires adequate space to place a screw around the vertebral artery. If a patient has an anomalous course of the vertebral artery, the Magerl technique can thus pose a potential risk of vessel injury. Lastly, the Magerl technique has a high risk of screw malpositioning and instrumentation failure [5].

More recently, Harms and Melcher described a stabilization technique based on fixation of the C1 lateral masses and C2 isthmus with polyaxial screws. This operation was first performed by Harms in August 1997, but was not published until 2001. Unlike the Magerl technique, the Harms technique overcomes neurovascular risk factors while providing equal biomechanical efficacy. More specifically, the Harm’s technique can be performed even if the posterior arch is involved (unlike with hook fixation), and/or loss of C1-C2 alignment occurs. Furthermore, the Harms technique has less risk of vertebral artery injury and provides a greater advantage of intraoperative reduction and fixation of atlantoaxial complex without instrumentation placement under the posterior arch of the C1 [4-6].

## Case presentation

A 79-year-old female presented with incapacitating chronic neck pain. The patient's neck pain was greater on the left side than the right and started gradually approximately 18 months ago. The patient described her neck pain as stabbing with 10/10 intensity. In addition to her neck pain, she complained of frequent headaches. She denied pain radiation down the left arm and denied history of any trauma. The patient was previously placed on physical therapy for six weeks and underwent a rhizotomy approximately a year ago. Neither physical therapy nor the rhizotomy provided long-term neck pain relief.

The patient’s medical history included carpal tunnel syndrome, arthritis, and cancer. Her surgical history included hip replacement, shoulder repair, and femur fracture repair. A physical examination showed a severely limited range of motion of the cervical spine, with pronounced limited range of cervical rotation to the left. The patient stated that her neck pain occasionally radiated into her skull. All other physical examinations were noncontributory.

**Figure 1:**
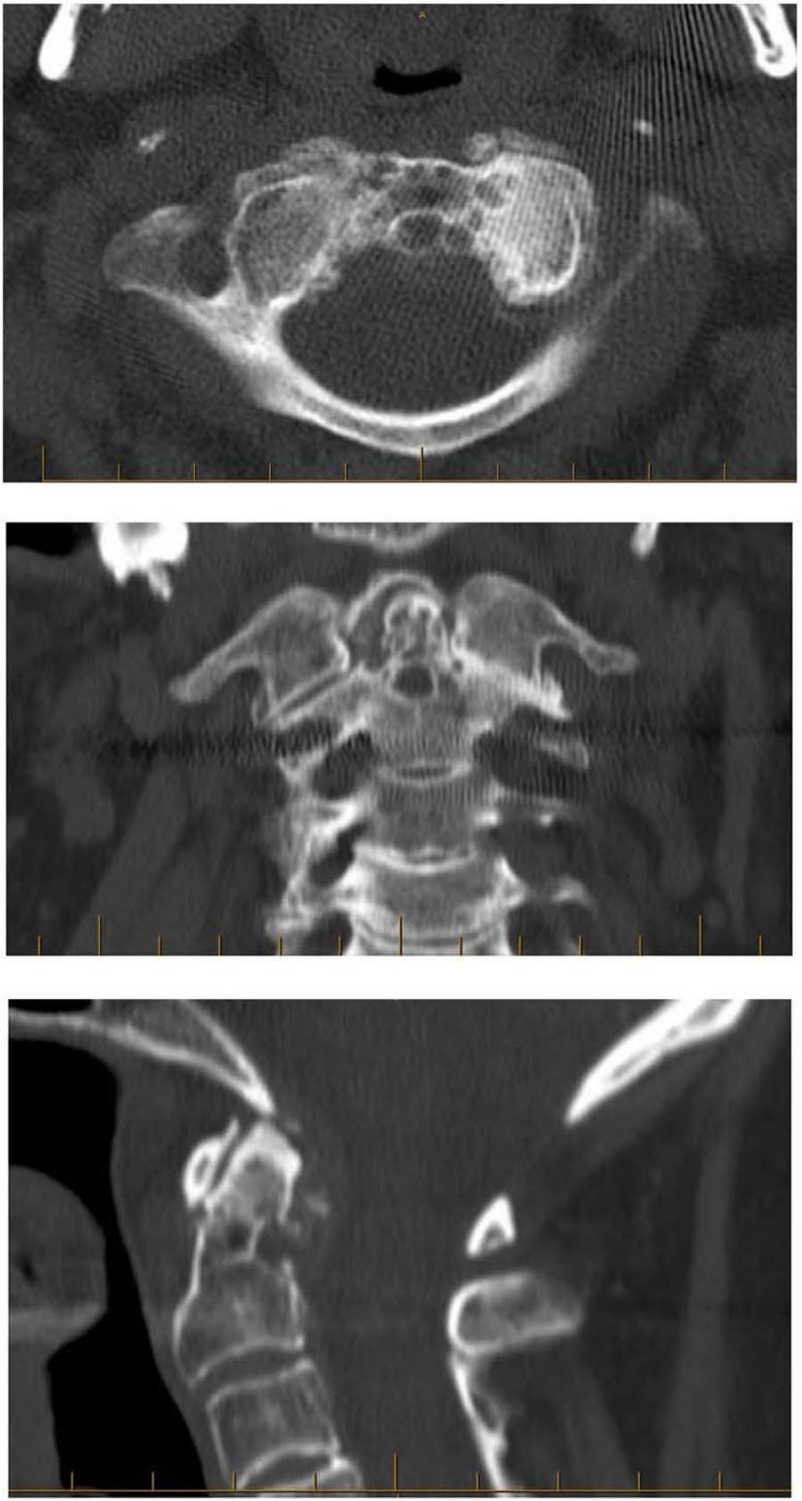
Preoperative images (CT scans)

**Figure 2:**
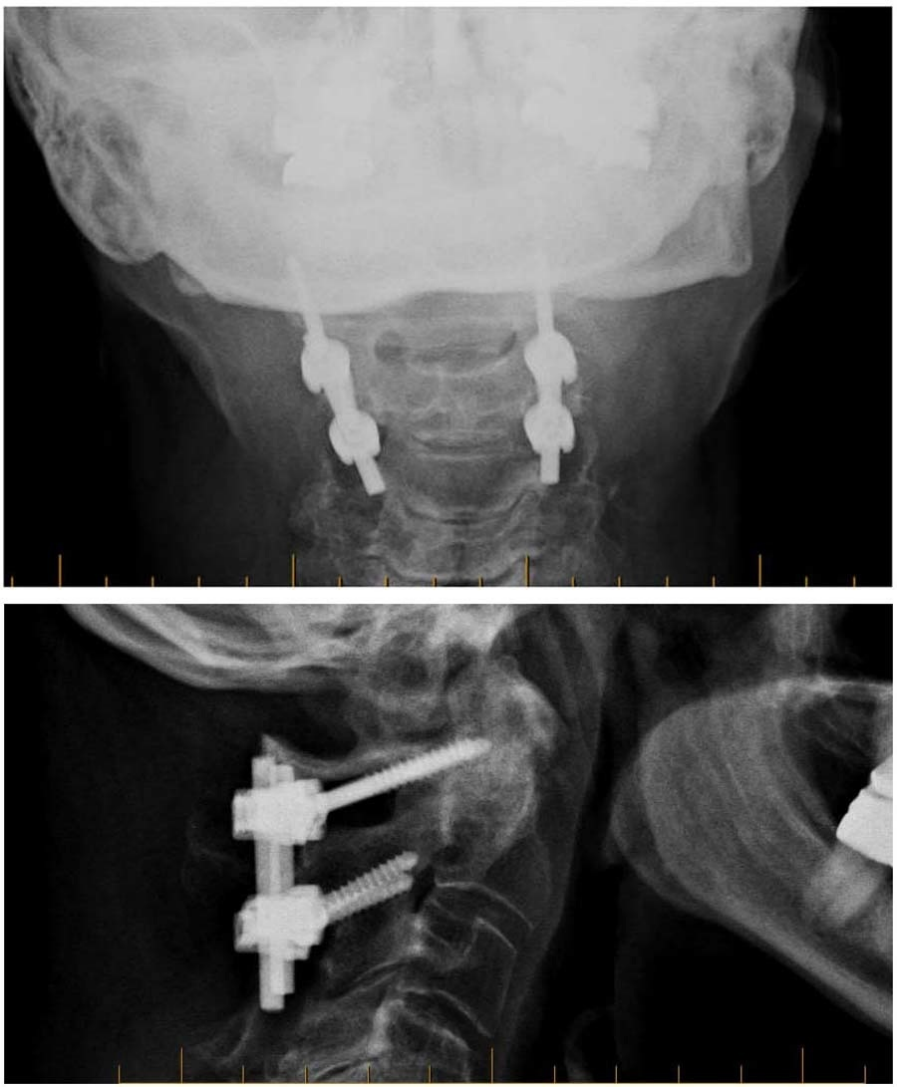
Postoperative images (X-rays)

A CT scan of the cervical spine showed severe left C1-C2 facet arthrosis with complete obliteration of the joint. The remainder of the spinal canal showed no significant spinal stenosis or any other damage that could explain her symptoms. The patient was recommended for a left C1-C2 facet joint injection before C1-C2 posterior fusion. After an epidural injection failed to deliver any pain relief, she underwent C1-C2 posterior fusion with Harms technique using iliac crest autogenous bone grafting. Post-surgery, a neck collar was prescribed for two months. Radiographs at the one-year follow-up showed intact instrumentation and solid C1-C2 fusion. The patient had resumed her daily activities, reported no pain, and was very happy with the procedure and its results. She denied having any recent conservative therapy. Preoperative and postoperative images are shown in Figure 1 and Figure 2, respectively. Informed consent was obtained from the patient for this study.

## Discussion

In 1984, Ehni and Benner explained the syndrome of lateral atlantoaxial osteoarthritis (AAOA). Ehni and Benner described AAOA as intractable neck pain that is often unilateral and radiates into the suboccipital region. Due to severe neck pain, people with AAOA experience limitation and difficulty with cervical rotation to the side. Conservative management of AAOA includes NSAIDs, bracing, chiropractic manipulations, physical therapy, intra-articular steroid injections, nerve blocks, rhizotomy, and/or C2 ganglionectomy [7-8]. Our patient had physical therapy for six months, a single epidural injection, and a rhizotomy procedure without any significant improvement in pain.

Over the years, many posterior techniques of C1-C2 fixation have been developed, and almost all are considered standard of care for atlantoaxial fusion. However, none of these techniques are without risks [9]. The Harms technique can reduce the risk of vertebral artery injury in comparison to transarticular screw fixation. Additionally, the advantages of the Harms technique include reduction of C1-C2, protection of the C1-C2 joint, and possibility of screw removal after regaining C1-C2 range of motion [10].

For this patient, we successfully used the Harms technique with iliac crest autogenous bone grafting with very satisfactory results of solid C1-C2 fusion and no reported neck pain at the one-year follow-up.

## Conclusions

We used the Harms technique with iliac crest autogenous bone grafting at the C1-C2 levels with successful solid fusion. At the one-year follow-up, the patient had resumed her daily activities, reported no pain, and was happy with the results.
